# Adaptive laboratory evolution of native methanol assimilation in *Saccharomyces cerevisiae*

**DOI:** 10.1038/s41467-020-19390-9

**Published:** 2020-11-04

**Authors:** Monica I. Espinosa, Ricardo A. Gonzalez-Garcia, Kaspar Valgepea, Manuel R. Plan, Colin Scott, Isak S. Pretorius, Esteban Marcellin, Ian T. Paulsen, Thomas C. Williams

**Affiliations:** 1 Department of Molecular Sciences, ARC Centre of Excellence in Synthetic Biology, Macquarie University, 2109 North Ryde, NSW Australia; 2 CSIRO Synthetic Biology Future Science Platform, Canberra, ACT 2601 Australia; 3 Australian Institute for Bioengineering and Nanotechnology, The University of Queensland, 4072 Brisbane, QLD Australia; 4 ERA Chair in Gas Fermentation Technologies, Institute of Technology, University of Tartu, 50411 Tartu, Estonia; 5 Queensland Node of Metabolomics Australia, AIBN, The University of Queensland, 4072 Brisbane, QLD Australia; 6 Biocatalysis and Synthetic Biology Team, CSIRO Land & Water, Black Mountain Science and Innovation Park, Canberra, Canberra, ACT Australia

**Keywords:** Metabolic engineering, Synthetic biology

## Abstract

Utilising one-carbon substrates such as carbon dioxide, methane, and methanol is vital to address the current climate crisis. Methylotrophic metabolism enables growth and energy generation from methanol, providing an alternative to sugar fermentation. *Saccharomyces cerevisiae* is an important industrial microorganism for which growth on one-carbon substrates would be relevant. However, its ability to metabolize methanol has been poorly characterised. Here, using adaptive laboratory evolution and ^13^C-tracer analysis, we discover that *S. cerevisiae* has a native capacity for methylotrophy. A systems biology approach reveals that global rearrangements in central carbon metabolism fluxes, gene expression changes, and a truncation of the uncharacterized transcriptional regulator Ygr067cp supports improved methylotrophy in laboratory evolved *S. cerevisiae*. This research paves the way for further biotechnological development and fundamental understanding of methylotrophy in the preeminent eukaryotic model organism and industrial workhorse, *S. cerevisiae*.

## Abstract (web summary)

Methylotrophic metabolism enables growth on methanol, an alternative to sugar fermentation. Here the authors use adaptive laboratory evolution to uncover native methylotrophy capacity in *Saccharomyces cerevisiae*.

## Introduction

Methylotrophic organisms are able to grow on one-carbon substrates such as carbon dioxide, carbon monoxide, methane, or methanol and are important primary producers for Earth’s ecosystems as they fix inorganic carbon into biologically available organic carbon. These organisms and their underlying metabolic networks are becoming increasingly important in global efforts to mitigate climate change and reduce our reliance on non-renewable resources such as fossil fuels. Our current dependence on fossil fuels is not sustainable owing to finite reserves and negative environmental impacts from extraction and use. By-products from fossil fuel combustion include a myriad of toxic air pollutants and CO_2_, which is the main anthropogenic contributor to climate change. These complex environmental problems call for a global effort to move towards a bio-economy in which microbial metabolism is used for the conversion of renewable materials into useful products^1^. Typically, sugars derived from sugarcane or corn are used as feedstocks for the production of fuels and chemicals from microbial metabolism. However, sugar production can be prohibitively expensive^2^ and requires arable land.

As an alternative to sugars, one-carbon (C1) substrates are abundant and can be obtained from natural gas or waste resources such as agricultural, municipal or industrial waste^3,4^. Methanol is a particularly attractive one-carbon substrate owing to its abundance and liquid state, which makes it more compatible with existing fermentation, storage and transportation infrastructure^3,5^. Methanol can also be obtained from methane and carbon dioxide stemming from industrial waste streams^6^. Engineering microorganisms to convert C1 compounds such as methanol into food, fuels and chemicals has therefore become a major goal in the field of synthetic biology^4,7^.

Methylotrophic metabolism enables energy and biomass generation from methanol and is present in bacterial, archaeal and yeast species. Recent attempts have been made to produce valuable metabolites using methylotrophs. For example, *Methylobacterium extorquens* AM1 has been engineered to produce 3-hydroxypropionic acid from methanol^8^. The methylotrophic yeast *Pichia pastoris* (renamed *Komagataella phaffii*^9^) is currently used industrially for production of recombinant proteins^10^. Recently, *P. pastoris* has also been used for production of metabolites including astaxanthin and isobutanol from sugar^11,12^. However, relative to model organisms, most native methylotrophs lack the genetic tools and depth of characterisation necessary for the successful metabolic engineering of high-yield and heterologous pathways. An attractive alternative to enable the use of methanol as feedstock is to engineer synthetic methylotrophy into industrially robust and well-characterised microorganisms. Model organisms are easier to genetically manipulate, providing opportunities to engineer non-native central carbon metabolism with greater genetic and metabolic plasticity. Given our understanding of their metabolism, model organisms are also more amenable to gaining fundamental insights. Recent approaches have focused on engineering synthetic methylotrophy in bacteria such as *Escherichia coli* and *Corynebacterium glutamicum*^13–20^. Incorporation of ^13^C-methanol into central carbon metabolites and specialty products has been demonstrated in both species, with growth on methanol as the sole carbon source recently engineered in *E. coli*^21^.

Methanol-utilisation research on model eukaryotes such as the yeast *Saccharomyces cerevisiae* has been limited, although *S. cerevisiae* has distinct advantages over organisms such as *E. coli* for use in industrial fermentation. For example, *S. cerevisiae* can correctly express, fold and post-translationally modify eukaryotic proteins, is not susceptible to phage contamination, has high tolerance to low pH concentrations and solvents like methanol, and has organelles that can be co-opted for localisation of specialised metabolism^22–24^. Previously, our work identified a methanol-specific transcriptomic response in the laboratory strain CEN.PK as well as methanol-specific growth. This response was stronger than that of the laboratory strain S288C. Thus, we sought to further characterise methanol metabolism in CEN.PK using adaptive laboratory evolution (ALE). Here, we identify the native capacity of *S. cerevisiae* to assimilate methanol into central carbon metabolism. Using methanol-dependent growth assays and ^13^C-methanol tracer studies, we characterise and improve native methanol assimilation in *S. cerevisiae*.

## Results

### Methanol-specific growth and 13C-methanol tracer analysis reveals native methanol assimilation in *S. cerevisiae*

**Fig. 1:**
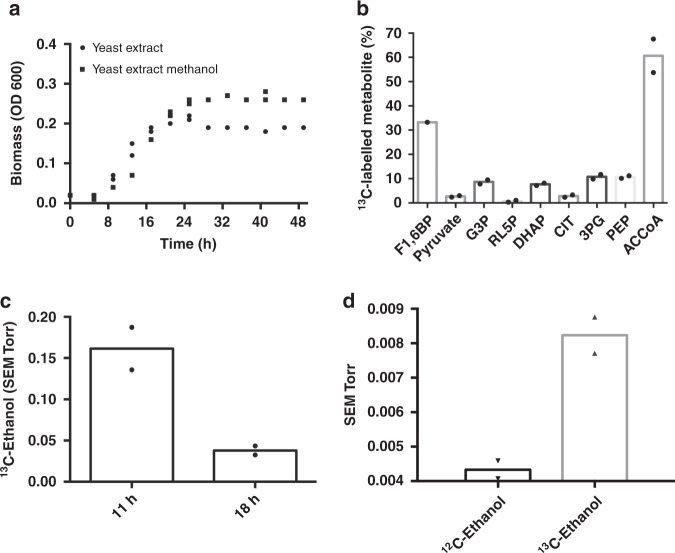
^13^C-methanol fermentations confirm methanol assimilation in *S. cerevisiae*. **a** Growth profile of CEN.PK 113-5D (with empty vector pRS416) grown in liquid YNB medium with 0.1% yeast extract (circles) or with 0.1% yeast extract and 2% methanol (squares). Data points represent the measurement of two independent biological replicates. Source data are provided as a Source Data file. **b** Percentage of fully ^13^C-labelled intracellular metabolite pool relative to total metabolite pool in CEN.PK 113-5D. *F1,6BP* fructose-1,6-bisphosphate, *G3P* glyceraldehyde-3-phosphate, *RL5P* ribulose-5-phosphate, *DHAP* dihydroxyacetone phosphate, *CIT* citrate, *3PG* 3-phosphoglyceric acid, *PEP* phosphoenolpyruvate, *ACCoA* acetyl-coenzyme A. Data points are from two independent biological replicates with bars representing the mean, except for CEN.PK 113-5D F1,6BP, for which no 12 C metabolite was detected in one replicate. **c** ^13^C-ethanol was produced by CEN.PK 113-5D. The signal intensity was normalised to the inert gas nitrogen, and then to biomass. Data show ^13^C-ethanol intensity at 47 amu for two independent biological replicates with bars representing the mean. **d** Comparison of ^12^C-ethanol and ^13^C-ethanol intensities during growth in ^13^C-methanol. The signal intensity was normalised to the inert gas nitrogen, and then to biomass for each strain. Data show the individual technical measurements for ^12^C-ethanol and ^13^C-ethanol intensity at 31 and 33 amu, respectively, for two independent biological replicates with bars representing the mean. *SEM*, secondary electron multiplier, *amu* atomic mass unit. Source data are provided as a Source Data file.

Our previous study compared two commonly used *S. cerevisiae* strains, S288C and CEN.PK 113-5D, for their transcriptional response and growth profile in the presence of methanol^25^. CEN.PK was identified as the superior candidate to engineer methylotrophy as it had better methanol tolerance and a distinct methanol-specific transcriptional response that included upregulation of genes involved in peroxisomal biogenesis (*PEX11*), alcohol oxidation (*ADH2*) and formate oxidation (*FDH1)*. Having identified the potential for methanol-dependent growth and metabolism in *S. cerevisiae*^25^, methanol-specific growth of CEN.PK 113-5D was further assessed using controlled bioreactor fermentations. Previous work with *E. coli* has highlighted the importance of yeast extract for growth on liquid methanol media^26^. Liquid yeast nitrogen base (YNB) medium with and without 2% methanol and 0.1% yeast extract was therefore used to assess the effect of methanol on growth (Fig. 1a). The presence of methanol in the medium resulted in a final OD_600_ increase of 39% relative to yeast extract-only medium, confirming a methanol-specific growth increase in liquid medium containing yeast extract.

To further investigate the methanol response in *S. cerevisiae* and determine the extent to which methanol enters central carbon metabolism in the presence of the co-substrate yeast extract, a ^13^C-methanol tracer analysis was performed. Bioreactor off-gases such as ^13^C-methanol, ^13^C-CO_2_, CO_2_, ^13^C-ethanol and ethanol were measured in real-time using a mass spectrometer connected to the bioreactors. Intracellular metabolites were analysed using Liquid Chromatography-Mass Spectrometry (LC-MS). ^13^C-ethanol (Fig. 1c, d) and fully ^13^C-labelled intracellular metabolites involved in the pentose phosphate pathway, glycolysis, and the TCA cycle were also detected (Fig. 1b). ^13^C-fructose-1,6-bisphosphate and ^13^C-pyruvate could only arise through the conversion of ^13^C-methanol through central carbon metabolism (Fig. 1b), confirming *S. cerevisiae* has a native capacity for methanol assimilation. In particular, the identification of a high proportion of fully ^13^C-labelled metabolite in the case of fructose-1,6-bisphosphate (33% of the metabolite pool) and of the structurally complex acetyl-CoA (60% of the metabolite pool) demonstrates methanol assimilation can occur in *S. cerevisiae*.

**Fig. 2:**
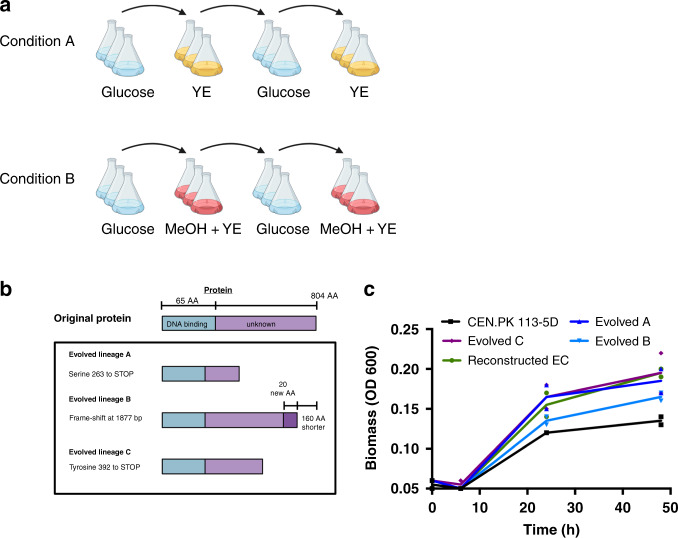
Adaptive laboratory evolution to improve native methanol assimilation in CEN.PK 113-5D. **a** Schematic of the ALE design. Three independent lineages of CEN.PK 113-5D (with pRS416) were grown in baffled shake flasks in condition A or B for 230 generations. Under condition A, cultures were passaged from YNB medium without amino acids and 1% glucose (24 h) to YNB medium without amino acids and 0.1% yeast extract (YE) (48 h). Under condition B, cultures were passaged from YNB medium without amino acids and 1% glucose to YNB medium without amino acids, 2% methanol and 0.1% yeast extract (MeOH + YE) (48 h). Figure was created with BioRender.com. **b** Schematic of the mutations in *YGR067C* from the three evolved lineages grown under condition B, and the changes they caused to the protein, all three mutations theoretically led to truncations. **c** Growth profiles of CEN.PK 113-5D (black squares), the three evolved lineages (A, dark blue upright triangles. B, light blue inverted triangles. C, purple diamonds.) in condition B, and the reconstructed CEN.PK 113-5D strain with the mutation observed in the evolved lineage C (green circles). Strains were grown in liquid YNB medium with 0.1% yeast extract and 2% methanol. Data are from two independent biological replicates with lines representing the mean. Source data are provided as a Source Data file.

### ALE of native methanol assimilation in *S. cerevisiae*

ALE was applied to characterise and improve native methanol metabolism in *S. cerevisiae*. The ALE strategy consisted of three independent lineages grown in 1× YNB medium with either yeast extract or yeast extract plus 2% methanol. Yeast extract-only medium was included to track any improvements in fitness specific to yeast extract. Cultures were grown in baffled shake flasks with alternating passages on 1% glucose or yeast extract with or without methanol (Fig. 2a). ALE relies on naturally occurring DNA replication errors to generate genetic diversity within a population. Owing to the relatively poor growth of *S. cerevisiae* on yeast extract methanol medium, pulsing between two different media was performed in order to increase the number of generations and mutations over time, while still exposing the population to methanol alongside a poor co-substrate (yeast extract) as a selection pressure. All six independent lineages were grown for 230 generations until a biomass (OD_600_) increase in yeast extract methanol medium was observed. The six evolved populations were re-sequenced to identify putative mutations leading to the phenotype. Only genes with mutations in all three methanol-exposed lineages that were absent in the yeast extract only lineages were considered. The only gene that matched these criteria was *YGR067C*, which had point mutations at different positions in the three methanol-evolved lineages (Fig. 2b). Ygr067cp is an uncharacterised putative transcription factor with a DNA-binding domain similar to that of *ADR1* (alcohol dehydrogenase II synthesis regulator). *ADRI* encodes a transcription factor that is involved in the expression of glucose-repressed genes, ethanol metabolism and peroxisomal proliferation^27,28^. All three independent mutations resulted in premature stop codons, which would result in truncation of the *YGR067C* protein (Fig. 2b). Mutations in the High Osmolarity Glycerol response gene *HKR1* (I584L) were identified in methanol-evolved lineages A and C, but not B, whereas a mutation in the cell wall protein encoding *FIT1* (V204A) gene was identified in all three yeast extract evolved lineages.

To test if truncation of the *YGR067C* transcription factor was responsible for improved growth on methanol, CRISPR-Cas9-mediated homologous recombination was used to introduce a stop codon in *YGR067C* of the wild-type strain as observed in the evolved lineage C (Fig. 2b). The mutation from evolved lineage C was chosen to reconstruct the phenotype as the truncation in Ygr067cp occurred midway between the evolved lineages A and B (Fig. 2b). This reconstructed CEN.PK 113-5D strain was referred to as reconstructed EC. The three evolved lineages, the parental CEN.PK 113-5D, and reconstructed EC strains were grown on yeast extract methanol medium to analyse growth differences (Fig. 2c). The evolved lineage A strain had a final biomass increase of 37% in the presence of methanol compared with the parental CEN.PK 113-5D strain, whereas the evolved lineage B and C strains showed a 22% and 44% increase, respectively. Importantly, the reconstructed EC strain has the same growth profile and final biomass increase (44%) as the evolved lineage C strain compared with the parental strain, indicating that the truncated transcription factor is responsible for increased growth in the presence of methanol. No growth improvement was observed between the parental and reconstructed EC strain on yeast extract only medium or minimal medium with glucose.

### Reconstructed EC strain characterisation and ^13^C-methanol tracer analysis

**Fig. 3:**
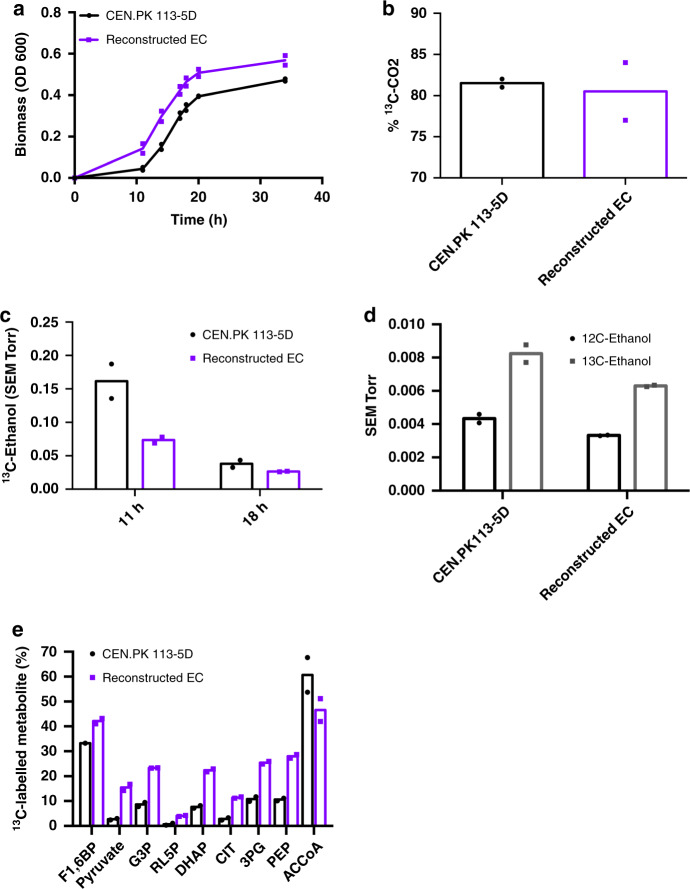
^13^C-methanol fermentations to characterise the reconstructed evolved strain. **a** Growth profile of CEN.PK 113-5D and the reconstructed evolved strain grown in liquid YNB medium with 2% ^13^C-methanol supplemented with 0.1% yeast extract cultures in bioreactors. Data are from two independent biological replicates with lines representing the mean. Source data are provided as a Source Data file. **b** Percentage of ^13^C-CO_2_/CO_2_ production in yeast extract ^13^C-methanol (2%) medium from two biological replicates with bars representing the mean. **c** ^13^C-ethanol was produced by CEN.PK 113-5D and by the reconstructed evolved strain. The signal intensity was normalised to the inert gas nitrogen, and then to biomass for each strain. Data show ^13^C-ethanol intensity at 47 amu for two independent biological replicates, with bars representing the mean. **d** Comparison of ^12^C-ethanol and ^13^C-ethanol intensities during growth in ^13^C-methanol. The signal intensity was normalised to the inert gas nitrogen, and then to biomass for each strain. Data show the ^12^C-ethanol and ^13^C-ethanol intensity at 31 and 33 amu, respectively, for two independent biological replicates, with bars representing means. *SEM* secondary electron multiplier, *amu* atomic mass unit. **e** Percentage of fully ^13^C-labelled intracellular metabolite pool relative to total metabolite pool of CEN.PK 113-5D and reconstructed evolved strains. *F1,6BP* fructose-1,6-bisphosphate, *G3P* glyceraldehyde-3-phosphate, *RL5P* ribulose-5-phosphate, *DHAP* dihydroxyacetone phosphate, *CIT* citrate, *3PG* 3-phosphoglyceric acid, *PEP* phosphoenolpyruvate, *ACCoA* acetyl-coenzyme A. Data are from two independent biological replicates, with bars representing means, except for CEN.PK 113-5D F1,6BP, for which no 12 C metabolite was detected in one replicate. Purple squares represent the Reconstructed EC strain, and black circles represent the CEN.PK 113-5D strain. Source data are provided as a Source Data file.

To characterise the effect that the reconstructed EC strain had on native methanol metabolism in *S. cerevisiae*, CEN.PK 113-5D and the reconstructed EC strain were grown in bioreactors with 2% ^13^C-methanol and 0.1% yeast extract. The reconstructed EC strain was chosen for ^13^C-methanol tracer analysis as it provides a clearer genotype–phenotype relationship as the only genotypic change between this strain and the parental CEN.PK 113-5D is the introduced truncation of Ygr067cp. Thus, any observed changes could most likely be attributed to the regulatory function of *YGR067C*. As previously noted (Fig. 2c), a growth advantage was observed in the reconstructed EC strain (Fig. 3a), which reached a higher final biomass compared with CEN.PK 113-5D. Both strains produced 80% of total CO_2_ as ^13^C-CO_2_ (Fig. 3b). However, the reconstructed EC strain produced less ^13^C-ethanol, particularly at 11 h (Fig. 3c, d). We hypothesised that the reconstructed EC strain could be redirecting methanol into biomass constituents and thus reducing ethanol production. ^13^C-methanol tracer analysis revealed that the reconstructed EC strain had a higher percentage of fully ^13^C-labelled intracellular metabolites compared with CEN.PK 113-5D (Fig. 3e). An approximate threefold increase in ^13^C-labelled glyceraldehyde-3-phosphate and dihydroxyacetone phosphate was observed as well as increased ^13^C-labelling of metabolites involved in lower glycolysis, including 3-phosphoglyceric acid, phosphoenolpyruvate, and pyruvate. ^13^C-labelled pyruvate increased from 2.7% in CEN.PK 113-5D to 15.4% in the reconstructed EC strain. Higher ^13^C-labelling was also observed for metabolites in the pentose phosphate pathway but the proportion of ^13^C-labelled metabolites was still low in both strains (<5%). An interesting observation was that CEN.PK 113-5D had a higher proportion of ^13^C-labelled acetyl-CoA compared with the reconstructed EC strain, 67.6% compared with 46.6% (Fig. 3e). Finally, no un-labelled or ^13^C-labelled ketoglutarate, fumarate, malate, oxaloacetate or glyoxylate were detected in the TCA cycle. The only metabolites that were detected in the TCA cycle were citrate and succinate, ^13^C-labelled citrate increased from 2.4% to 11.4% in the reconstructed EC strain while the same levels (0.5%) were observed in both strains for ^13^C-labelled succinate.

**Fig. 4:**
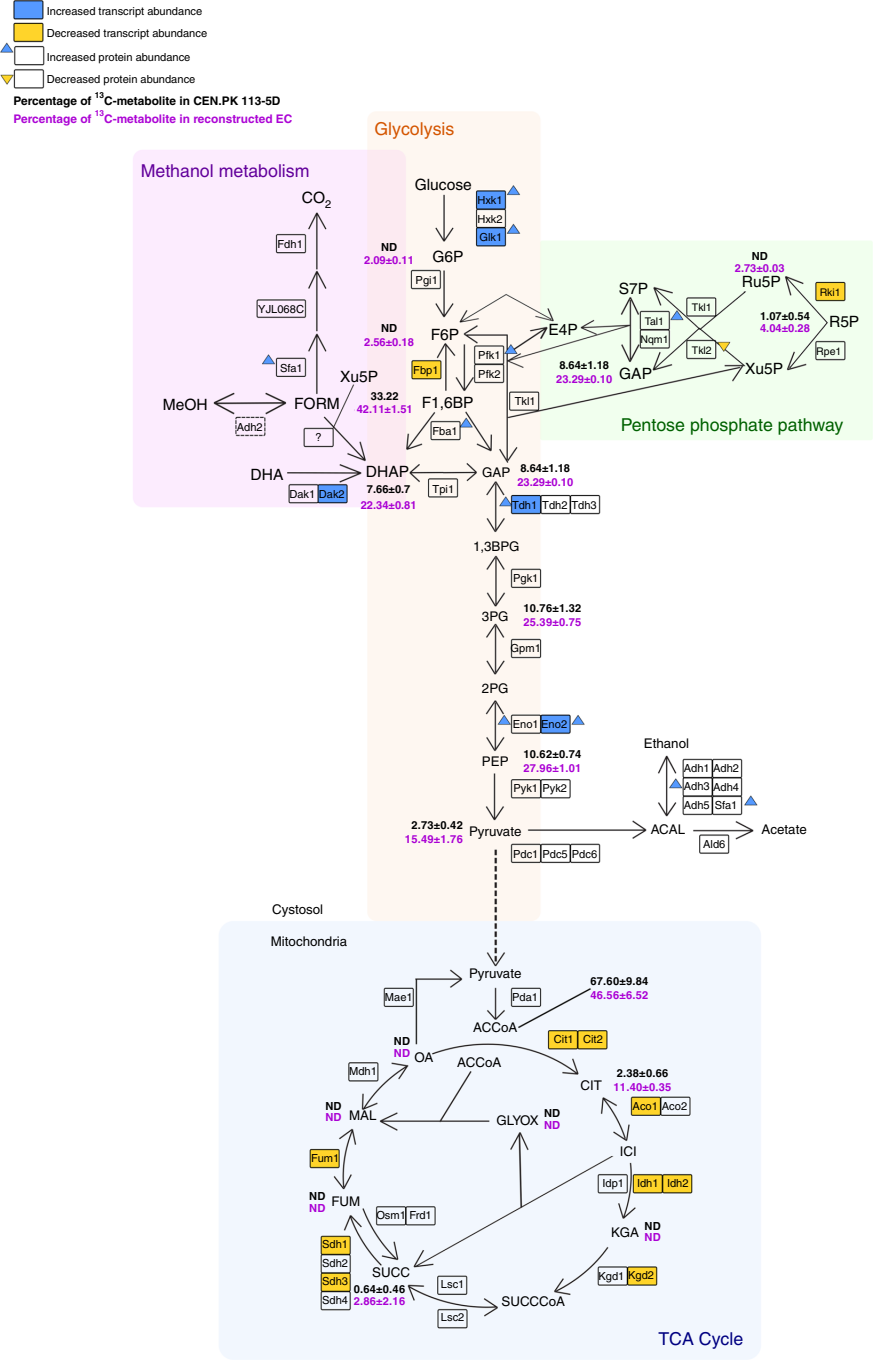
Characterisation of the reconstructed evolved strain at the metabolite, transcriptome and proteome level compared with the parental strain during ^13^C-methanol fermentations. Percentage of ^13^C-labelled intracellular metabolites in CEN.PK 113-5D (black) and the reconstructed evolved strain (purple). Metabolites are fully labelled with ^13^C. Data points represent the average of two independent biological replicates with standard deviation. Transcript and protein abundance in the reconstructed evolved strain compared with the parental strain. Genes with up- or downregulated fold-changes and adjusted *p* values <0.01 are coloured blue or yellow, respectively. Increased or decreased protein abundance and adjusted *p* values <0.05 is shown in blue or yellow arrows, respectively. *ND* not detected. The metabolic map was redrawn based on ref. ^58^. Metabolite abbreviations: *MeOH* methanol, *FORM* formaldehyde, *DHA* dihydroxyacetone, *DHAP* dihydroxyacetone phosphate, *Xu5P* xylulose-5-phosphate, *G6P* glucose-6-phosphate, *F6P* fructose-6-phosphate, *F,6BP* fructose-1,6-bisphosphate, *GAP* glyceraldehyde-3-phosphate, *1,3BPG* 1,3-bisphosphoglycerate, *3PG* 3-phosphoglycerate, *2PG* 2-phosphoglycerate, *PEP* phospheonolpyruvate, *OA* oxaloacetate, *CIT* citrate, *SUCCCoA* succinyl-coenzyme A, *ACCoA* acetyl-coenzyme A, *ICI* isocitrate, *AKG* alpha-ketoglutarate, *SUCC* succinate, *FUM* fumarate, *MAL* malate, *GLYOX* glyoxylate, *ACAL* acetaldehyde, *6PGL* 6-phospho-d-glucono-1,5-lactone, *6PGC* 6-Phospho-d-gluconate, *Ru5P* ribulose-5-phosphate, *R5P* ribose-5-phosphate, *S7P* sedoheptulose-7-phosphate, *E4P* erythrose-4-phosphate. Enzyme abbreviations: alcohol dehydrogenase (Adh1, Ahd2, Ahd3, Adh4, Adh5, SFA1), formate dehydrogenase (Fdh1), aldehyde dehydrogenase (Ald6), pyruvate decarboxylase (Pdc1, Pdc5, Pdc6), dihydroxyacetone kinase (Dak1, Dak2), triose phosphate isomerase (Tpi1), glyceraldehyde-3-phosphate dehydrogenase (Tdh1, Tdh2, Tdh3), fructose-1,6-bisphosphate aldolase (Fba1), phosphoglucose isomerase (Pgi1), hexokinase (Hxk1, Hxk2, Glk1), phosphofructokinase (Pfk1, Pfk2), transketolase (Tkl1, Tkl2), 3-phosphoglycerate kinase (Pgk1), phosphoglycerate mutase (Gpm1), enolase (Eno1, Eno2), pyruvate kinase (Pyk1, Pyk2), pyruvate dehydrogenase (Pda1), citrate synthase (Cit1, Cit2), aconitase (Aco1, Aco2), isocitrate dehydrogenase (Idh1, Idh2, Idp1), alpha-ketoglutarate dehydrogenase (Kgd1, Kgd2), succinyl-CoA ligase (Lsc1, Lsc2), fumarate reductase (Osm1, Frd1), succinate dehydrogenase (Sdh1, Sdh2, Sdh3, Sdh4), fumarase (Fum1), malate dehydrogenase (Mdh1), malic enzyme (Mae1), transaldolase (Tal1, Nqm1), formaldehyde dehydrogenase (Sfa1), d-ribulose-5-phosphate 3-epimerase (Rpe1), Ribose-5-phosphate ketol-isomerase (Rki1). Source data are provided as a Source Data file.

To further characterise the changes in the reconstructed EC strain, global transcript and protein levels were compared with the parental strain (Fig. 4). During growth on 2% ^13^C-methanol and 0.1% yeast extract, 243 transcripts were found to be significantly differentially expressed in the reconstructed EC strain (adjusted *p* < 0.01) with 111 genes downregulated and 132 genes upregulated (Supplementary File 1). Gene-list analysis of the downregulated genes showed Gene Ontology (GO) pathway enrichment of the TCA cycle, respiration and the glyoxylate cycle. No pathway enrichment was found for the upregulated genes in the reconstructed EC strain but GO process enrichment was found for ‘carbohydrate transmembrane transport’, ‘carbohydrate metabolic process’, ‘glycolytic process’ and ‘pyruvate metabolic process’, among others. Proteomics analysis showed 103 proteins were significantly altered in abundance in the reconstructed EC strain relative to the parent (adjusted *p* < 0.05; Supplementary File 2) with 84 proteins increased and 19 proteins decreased. Gene-list analysis of proteins with increased expression showed pathway enrichment of the ‘superpathway of glucose fermentation’, whereas the proteins with decreased abundance showed GO process enrichment only for ‘trehalose metabolic process’.

Both transcriptomics and proteomics indicated there were significant changes in central carbon metabolic pathway expression between the evolved reconstructed and parental strain. Five genes involved in glycolysis had higher transcript abundance in the reconstructed EC strain compared with CEN.PK 113-5D (*HXK1*, *GLK1*, *DAK2*, *TDH1* and *ENO2*), whereas *FBP1*, coding for fructose-1,6-bisphosphatase, a key enzyme involved in gluconeogenesis^29,30^, had lower transcript abundance. Hxk1p, Glk1p, Tdh1p and Eno2p also had increased protein abundance, as well as Pfk1p, suggesting glycolysis rather than gluconeogenesis is predominantly occurring during growth on methanol in the reconstructed EC strain. The increased transcript abundance of the 6-phosphofructo-2-kinase glycolysis regulator *PFK27*^31^ in the reconstructed EC strain also supports this concept. Concordant with the metabolite profile of the reconstructed EC strain, lower transcript abundance was observed for nine genes involved in the TCA cycle (*CIT1, CIT2*, *ACO1*, *IDH1*, *IDH2*, *KGD2*, *SDH1*, *SDH3* and *FUM3*; Fig. 4), suggesting the TCA cycle is downregulated during growth on methanol and yeast extract. Sfa1p, which is involved in native formaldehyde detoxification and alcohol oxidation also had increased protein abundance in the reconstructed EC strain. Finally, the pentose phosphate pathway showed interesting results, with *RKI1* having lower transcript abundance and Tkl2p lower protein abundance, whereas Tal1p and Fba1p had higher protein abundance.

### *ADH2* and *ACS1* deletion reduces methanol-specific growth

Four genes were selected to analyse their potential role in native methanol metabolism in the reconstructed EC strain. First, the gene coding for an alcohol dehydrogenase 2 (*ADH2*) was selected as in a separate study it was significantly upregulated in response to methanol^25^ and owing to the promiscuity of alcohol dehydrogenases in *S. cerevisiae*, it could be oxidising methanol to formaldehyde. *CAT8*, coding for a transcription factor involved in de-repressing genes during growth on non-fermentable carbon sources and thought to regulate the mutated transcription factor Ygr067cp^32^ (Fig. 2b) was chosen to analyse its effect on methanol growth. The serine hydroxymethyltransferase gene (*SHM1*) was chosen for deletion as another possibility for C1 carbon assimilation in *S. cerevisiae* involves formaldehyde detoxification to formate and then assimilation through the glycine cleavage complex^33^. Shm1p is responsible for converting serine to glycine and 5,10-methylenetetrahydrofolate and would be required if biomass formation was due to formaldehyde detoxification and subsequent formate assimilation. Finally, *ACS1* coding for an Acetyl-CoA synthetase was selected as a study by Oshawa et al.^34^ analysed the role of *ACS1* in *P. pastoris* and found that it is involved in ethanol repression, which is needed for the expression of methanol-induced genes.

**Fig. 5:**
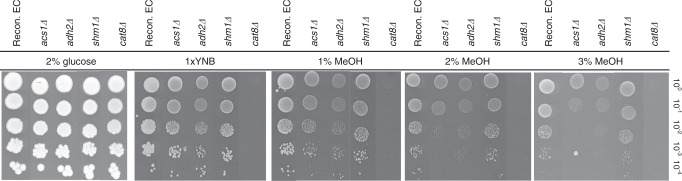
Growth in methanol of different gene deletions to test their putative involvement *in S. cerevisiae*’s native methanol assimilation. Growth on solid 1×YNB medium with different carbon sources was tested using serial ten-fold dilutions of the reconstructed evolved strain with an empty vector or *ACS1, ADH2*, *SHM1* or *CAT8* deletions. Yeast nitrogen base (YNB), yeast extract (YE), methanol (MeOH). Images were taken after incubating at 30 °C for 6 days. Images are representative of two repeated experiments.

Single deletion strains of the aforementioned genes were constructed in the reconstructed EC strain and tested by spotting the strains onto solid minimal YNB medium with 2% glucose, no additional carbon source, or increasing methanol concentrations (Fig. 5). The reconstructed EC strain *Δadh2* grew similarly to the reconstructed EC strain in minimal medium with 2% glucose or no additional carbon source (1× YNB) but growth was dramatically reduced on media containing methanol (1–3%), suggesting it is needed for methanol oxidation to formaldehyde. When *Δcat8* was deleted from the reconstructed EC strain, growth was only observed with 2% glucose as the carbon source, and its deletion dramatically decreased growth on methanol and 1× YNB, as previously reported^35^. Any potential role of *CAT8* in methanol assimilation could therefore not be analysed using this method. Deletion of *SHM1* had no effect on growth compared with the reconstructed EC strain on media with either glucose or methanol, suggesting it is not involved in the native methanol assimilation pathway. Interestingly, *ACS1* deletion showed no growth effect with 1% methanol, but had almost no growth when methanol was present at higher concentrations (2 and 3%).

## Discussion

Methanol is emerging as an important C1 feedstock for industrial biotechnology, making the characterisation and improvement of native methanol metabolism in model organisms critical to utilising this feedstock. Here, we analysed native methanol utilisation in *S. cerevisiae* CEN.PK 113-5D. First, a methanol-specific growth increase was seen in medium containing low amounts of yeast extract as a co-substrate (Fig. 1a). ^13^C-methanol trace analysis was then used to analyse the methanol metabolism of CEN.PK 113-5D. ^13^C-ethanol and ^13^C-labelled intracellular metabolites were produced (Fig. 1b, c), demonstrating methanol was not only being utilised by CEN.PK but also assimilated through central carbon metabolism. Importantly, *S. cerevisiae* has never been classified as a methylotroph, and no previous growth on- or assimilation of- methanol has been recorded in the literature. The identification of fully ^13^C-labelled metabolites from ^13^C-methanol clearly showed that methanol can be assimilated through central carbon metabolism in *S. cerevisiae*.

After identifying native methanol assimilation, we sought to characterise and optimise this pathway using ALE (Fig. 2a). Laboratory evolution experiments have been successful in optimising consumption of C1 carbon sources such as methanol and CO_2_ in *E. coli*, and CO_2_ in *P. pastoris*^14,36,37^. ALE strategies are also helpful for elucidating non-obvious biological engineering solutions and understanding partially characterised systems. In this study, final biomass was increased in liquid yeast extract methanol medium by 44% (Fig. 2a) after 230 generations. Whole-genome sequencing revealed mutations in an uncharacterised putative transcription factor *YGR067C* that led to truncations of the protein (Fig. 2b). Ygr067cp truncation was reverse engineered into the parental CEN.PK 113-5D strain, and the phenotype was restored (Fig. 2c), confirming that mutations in *YGR067C* are responsible for the improved growth on methanol. Ygr067cp shares an identical DNA-binding domain with Adr1p and is regulated by Cat8p^28,32^. Both Adr1p and Cat8p are involved in de-repression of genes needed for growth on non-fermentable carbon sources such as ethanol or glycerol^32^. Adr1p also activates genes involved in peroxisomal biogenesis and proliferation^28^. Genes under the control of Adr1p that are relevant to methanol metabolism include formate dehydrogenases (*FDH1*/*FDH2*), *ADH2*, catalase (*CTA1*) and *PEX11*, a peroxisomal membrane protein^28^. The truncation of Ygr067cp could have caused the constant expression of genes that are normally repressed, leading to more favourable regulatory conditions and metabolic fluxes for growth on methanol.

To characterise the improved growth on methanol, the reconstructed EC strain was grown on ^13^C-methanol and compared with the parental strain CEN.PK 113-5D (Fig. 3). The reconstructed EC strain had an improved capacity for methanol assimilation, with higher proportions of ^13^C-labelled intracellular metabolites observed (Fig. 3e). Moreover, integrated metabolomics, transcriptomics and proteomics analyses showed that the reconstructed EC strain has a different metabolic profile compared to the parent, with the TCA cycle and gluconeogenesis enzymes being downregulated (Fig. 4). Together with the upregulated genes (*DAK2, TDH1* and *ENO2*) and higher protein abundance of Pfk1p, Fba1p, Tdh1p and Eno2p, it is likely that a net glycolytic flux increase occurs during methanol assimilation in the reconstructed EC strain relative to the parental control strain.

The downregulated transcripts and proteins that we observed in the TCA and glyoxylate cycles fully overlap with those that are normally de-repressed during growth on non-fermentable carbon sources in a *CAT8-* and *SNF1-*dependent manner^28,32^. Given that *CAT8* is known to regulate *YGR067C*^32^, it is likely that truncation of the *YGR067C* protein facilitates a decoupling of methanol assimilation from the traditional non-fermentable carbon source utilisation phenotype in yeast. At present, it is unclear how these metabolic rearrangements favour methylotrophy in *S. cerevisiae*. One possibility is that glycolytic rather than gluconeogenic fluxes favour the pentose phosphate pathway fluxes necessary for methanol assimilation in the presence of yeast extract. Another point worth noting is that many obligate methylotrophs operate an incomplete, downregulated TCA cycle through the absence of α-ketoglutarate dehydrogenase activity, which is thought to preclude heterotrophic growth^38^.

It is unclear how formaldehyde, the oxidation product of methanol, is assimilated in *S. cerevisiae*. However, the high levels of ^13^C-labelling we saw in dihydroxyacetone phosphate, fructose-1-6-bisphosphate, and glyceraldehyde-3-phosphate suggest assimilation occurs via a mechanism similar to the *P. pastoris* XuMP pathway where formaldehyde and xylulose-5-phosphate are converted into dihydroxyacetone and glyceraldehyde-3-phosphate. Based on the higher transcript abundance of Tal1p and Fba1p, it is possible that the non-oxidative branch of the pentose phosphate pathway is going through different rearrangement reactions than those when *S. cerevisiae* is grown on glucose to recycle the co-substrates needed for formaldehyde assimilation. Transaldolase could be catalysing the rearrangements of fructose-6-phosphate and sedoheptulose-7-phosphate to glyceraldehyde-3-phosphate and erythrose-4-phosphate as it was recently postulated in *P. pastoris* grown on methanol^39,40^. To identify specific genes involved in native methanol assimilation, *ADH2* and three other genes were deleted from the reconstructed EC strain. Another study from our group found that *ADH2* was upregulated when *S. cerevisiae* was grown in the presence of methanol^25^ and when the reconstructed EC strain with an *ADH2* deletion was grown on solid minimal media with increasing concentrations of methanol, growth was severely hindered in all cases, suggesting its role in oxidising methanol to formaldehyde (Fig. 5). We also observed that deletion of acetyl-CoA synthetase (*ACS1*) reduced growth on solid methanol media (Fig. 5). Acs1p is important for growth on non-fermentable carbon sources^41^, and its homologue is an important regulator of methylotrophic metabolism in *P. pastoris*^34,42^. Acs1p is therefore likely to be an important source of acetyl-CoA in *S. cerevisiae* methanol metabolism.

Native methanol assimilation was identified in *S. cerevisiae* and improved through ALE, providing the possibility of exploring native methanol metabolism. Higher biomass formation, a complete understanding of native methylotrophic metabolism, and the elimination of the requirement for yeast extract in liquid methanol medium are the most immediate challenges remaining for the implementation of growth with methanol as the sole carbon source in *S. cerevisiae*. After robust methylotrophy is established in *S. cerevisiae*, existing metabolite production pathways could potentially be coupled to a methanol converting ‘platform strain’ using the state-of-the-art genetic tools and the deep physiological characterisation available in this model organism. Together, the results from this work represent an exciting step towards using sustainable feedstocks during microbial fermentations for conversion to chemicals, fuels, materials, pharmaceuticals and foods, and provide a glimpse of an unexplored metabolic network in a model eukaryote.

## Methods

### Strains and plasmids

*S. cerevisiae* plasmids and strains used in this study are shown in Tables 1 and 2, respectively. All strains are available from the authors upon request. To delete *ADH2*, *CAT8*, *SHM1* and *ACS1* from the reconstructed EC strain, the orf::KanMX locus from the BY4741 knockout collection^43^ plus 500 bp up- and downstream was PCR amplified using primers 1–8. Amplified constructs were then transformed into the reconstructed EC strain and plated on yeast extract peptone dextrose (YPD) with Geneticin (200 μg/mL; Gibco™ 10131035). Independent colonies were screened via PCR using primers 9–16 that annealed 200 bp outside of the PCR-generated homology region. All primers and their sequences are listed in Supplementary Table 3.

**Table 1:** Plasmids used in this study.

Name	Details	Origin
pRS416	Yeast centromeric plasmid, URA3 marker	Euroscarf^57^

**Table 2:** *Saccharomyces cerevisiae* strains used in this study.

Name	Genotype, plasmids	Notes	Origin
CEN.PK 113-5D	MATa; *ura3-52*	Haploid laboratory strain with uracil auxotrophy, mating type ‘a’	Euroscarf^57^
CEN01	CEN.PK 113-5D, pRS416	Prototrophic strain with empty *URA3* vector	This study
REC. EC	CEN.PK 113-5D with modified *YGR067C*^Y392*^, pRS416	Strain expressing a truncated Ygr067cp where Tyrosine at position 392 is replaced with a stop codon	This study
REC. EC *adh2Δ*	CEN.PK 113-5D with modified *YGR067C*^Y392*^, pRS416	Strain expressing a truncated Ygr067cp where Tyrosine at position 392 is replaced with a stop codon, and Adh2p deletion	This study
REC. EC *shm1Δ*	CEN.PK 113-5D with modified *YGR067C*^Y392*^, pRS416	Strain expressing a truncated Ygr067cp where Tyrosine at position 392 is replaced with a stop codon, and Shm1p deletion	This study
REC. EC *cat8Δ*	CEN.PK 113-5D with modified *YGR067C*^Y392*^, pRS416	Strain expressing a truncated Ygr067cp where Tyrosine at position 392 is replaced with a stop codon, and Cat8p deletion	This study
REC. EC *acs1Δ*	CEN.PK 113-5D with modified *YGR067C*^Y392*^, pRS416	Strain expressing a truncated Ygr067cp where Tyrosine at position 392 is replaced with a stop codon, and Acs1p deletion	This study

### Media and growth conditions

*E. coli* DH5α cells were used for plasmid propagation/storage and grown in lysogeny broth media (1% tryptone, 0.5% yeast extract, 1% NaCl) with ampicillin.

The strains were precultured on yeast nitrogen base (YNB) medium without amino acids and with 5 g/L ammonium sulphate (Sigma-Aldrich Y0626) and 10 g/L glucose. Growth experiments were performed on YNB medium with 1 or 2% methanol supplemented with 1 g/L yeast extract (Merck 103753). Optical density readings at 600 nm (OD_600_) were used to track growth. For spot assays, swabs from streaked agar plates were precultured twice in 10 mL of 1× YNB, 1% glucose in sterile 50 mL Falcon tubes. During the exponential phase of the final pre-culture, cells were washed twice in 10 mL of sterile MilliQ water and serially diluted 10-fold up to 10^−4^, prior to spotting 5 μl from each dilution onto the indicated agar plates. Plates were incubated at 30 °C for 6 days. Spot assay photos are representative of repeated experiments. For the ALE experiment, three independent lineages of CEN.PK 113-5D with pRS416 where grown on baffled shake flasks with 50 mL YNB medium containing 1 g/L yeast extract or 1 g/L yeast extract with 2% methanol (48 h) pulsing with YNB medium with 1% glucose (24 h), and incubated at 30 °C. OD_600_ measurements were taken before every passage and the cultures were inoculated at a starting OD_600_ of 0.2 if grown on conditional medium or OD_600_ of 0.02 if grown on glucose medium.

### DNA extraction and whole-genome sequencing of ALE lineages

Glycerol stocks of the six independent evolutionary lineages were inoculated into 10 mL of YPD medium and grown overnight. Cells were pelleted and washed twice in 10 mL sterile MilliQ water by centrifuging at 4000 × *g* for 2 mins. Genomic DNA was extracted from pellets using the Yeast DNA Extraction Kit from ThermoFisher (catalogue number 78870) according to the instructions. Whole-genome sequencing was performed at the Ramaciotti Centre for Genomics using Nextera XT library preparation and NextSeq500 2 × 150 bp sequencing. A minimum of 11 million reads were generated per sample with >95% of reads having Q20 quality scores, except for methanol-evolved lineage B, which had 80% of reads with at least Q20. An annotated CEN.PK 113-7D reference genome was generated by transferring annotated coding sequences with greater than 95% homology from the S288C reference genome to CEN.PK 113-7D FASTA files using Geneious Pro version 11^44^. Untrimmed reads were mapped to the annotated CEN.PK 113-7D reference genome using Geneious Pro. Each sample had >99% of reads mapped to the reference with ~100-fold coverage. Non-synonymous single nucleotide variants (SNV) within coding sequences were identified at a minimum coverage of 10×, read-variant frequency of 0.9, maximum variant *p* value of 10^−5^, and a minimum strand-bias *p* value of 10^−5^ when exceeding 65% bias. SNVs from each evolved lineage were annotated onto the same CEN.PK reference sequence and inspected visually using Geneious Pro. SNVs present in the three methanol-exposed lineages but absent in the three yeast extract only lineages were identified as potentially causative mutations. Mutations present in all six lineages were assumed to be present in the parental CEN.PK 113-5D strain, and were not considered any further. Raw genome sequencing reads have been deposited at the National Centre for Biotechnology Information under Bioproject number PRJNA612896.

### Reconstruction of evolved lineage C mutation in *YGR067C*

The methanol-exposed evolutionary lineages had convergent but non-identical mutations in *YGR067C*, which all encoded truncations in the protein through premature stop codons. These mutations were substitution of C to G at nucleotide position 788, deletion of A at position 1877 and substitution of C at position 1176 to G. The C1176G mutation from lineage C was chosen for reverse engineering into the parental CEN.PK 113-5D strain owing to the proximity of the mutation to a favourable CRISPR-Cas9 PAM site. The mutation was constructed using a single pRS423 plasmid encoding the Cas9 protein, the guide RNA and a hygromycin selection marker, which is PCR amplified using primers that encode a new guide sequence in their 5’ extensions (primers 17 and 18) and have 20 nt of homology to one another^45–47^. This PCR product was co-transformed with mutated *YGR067C* DNA that was PCR amplified from evolved lineage C genomic DNA using primers 19 and 20, with transformants selected for hygromycin resistance on YPD agar plates. Crude genomic DNA was extracted using the lithium acetate-sodium dodecyl sulphate (SDS) method where colonies are heated for 5 min at 70 °C in 100 µL of 200 mM lithium acetate, 1% SDS prior to DNA precipitation using 100% ethanol, pelleting at 20,000 × *g* and resuspension in 100 µL of sterile water^48^. Transformants were screened by PCR amplifying and Sanger sequencing the putatively mutated region of *YGR067C* using primers 21 and 22, which anneal outside the *YGR067C* locus. One out of 12 tested colonies had the desired mutation using this method. This strain was subsequently referred to as ‘reconstructed EC’. All primers and their sequences are listed in Supplementary Table 3.

### ambr® 250 bioreactor fermentations

CEN.PK 113-5D was precultured as above in sterile 50 mL Falcon tubes prior to inoculation at an OD_600_ of 0.02 in ambr^®^ 250 (Sartorius Stedim) microbial bioreactors with 140 mL of medium. Dissolved oxygen was maintained at a minimum level of 20% via automatic control of stirring speed, air sparging and O_2_ sparging. pH was maintained at five via the automatic addition of 5 M potassium hydroxide or 1 M phosphoric acid, and temperature was kept at 30 °C. Bioreactors were sampled robotically at indicated time points, with 1 mL samples stored at −20 °C prior to measurement of growth using OD_600_.

### 13C-methanol fermentations

Growth experiments in YNB medium supplemented with yeast extract (1 g/L) and ^13^C-methanol (2%; Sigma-Aldrich 277177) were carried out aerobically (sparging air) at 30 °C, agitation of 300 RPM, and with a starting volume of 250 mL of medium using the Multifors 2 bioreactor system (Infors AG). pH was maintained between 4.8 and 5.2 using 3 M H_3_PO_4_. For pre-culturing, biological replicates of each strain were grown in liquid YNB medium for ~15 h. Cultures were then passaged into a second pre-culture and grown to mid-exponential phase (OD_600nm_ between 1 and 3). A third pre-culture was also grown to mid-exponential phase and washed twice with sterile water before inoculating the experimental cultures at an OD_600_ of 0.02.

### RNA extraction, sequencing and transcriptome analysis

RNA samples were taken at 34 h during the ^13^C-methanol (2%) fermentations. In all, 30 mL of culture was centrifuged at 4 °C 17,000 × *g* for 10 min, with pellets resuspended in RNA later (Sigma-Aldrich R0901) and stored at −20 °C prior to RNA extraction. Total RNA was extracted by digesting cell pellets in five units of zymolyase in the digestion buffer of the YeaStar RNA extraction kit (Zymo Research catalogue number R1002) for 1 h at 37 °C, followed by column purification using the RNeasy Plus Mini Kit (QIAGEN catalogue number 74136). Library preparation and sequencing was performed at the Ramaciotti Centre for Genomics using a TruSeq Stranded mRNA-seq preparation kit and NextSeq 500 2×75 bp sequencing. Thirty million reads were generated per sample with Q30 > 93% and Q20 > 95%. Untrimmed reads were mapped to the S288C genome with read counting and differential expression analysis carried out using Geneious Pro version 11^44^ by mapping reads to the S288C reference genome using the Geneious RNA algorithm with default settings. RPKM, FPKM and TPM read counts were calculated prior to differential expression analysis using the DeSeq2^49^ within Geneious, and a false discovery rate (FDR) of 0.1. Approximately 72 million reads were generated per sample. Genes with adjusted *p* values <0.01 were considered differentially expressed. Lists of up and downregulated genes were analysed for GO term and pathway enrichment using YeastMine at the Saccharomyces Genome Database (https://yeastmine.yeastgenome.org/yeastmine/bag.do). Significant enrichment against the background whole-genome gene list was identified with p-values less than 0.05 and Holm–Bonferroni correction for multiple comparisons. A list of all significant genes that were differentially expressed can be found in Supplementary File 1.

### Off-gas data analysis

Real-time analysis of bioreactor culture off-gas was achieved using a Hiden HPR-20-QIC mass spectrometer (Hiden Analytical) that was connected to the bioreactors. The Faraday Cup detector was used to monitor the signal intensities of N_2_, Ar, CO_2_, ^13^C-CO_2_, ethanol, ^13^C-ethanol and ^13^C-methanol at 28, 40, 44, 45, 27, 47 and 30 atomic mass unit (amu), respectively. To increase sensitivity and detect the presence of ^13^C-ethanol from the off-gas data, the Secondary Electron Multiplier (SEM) detector was used to scan any intensities from 15 to 50 amu, with 47 and 48 amu corresponding to ^13^C-ethanol. N_2_ intensity (constant during fermentation as nitrogen was an inert gas in our experiments) at 28 amu was used to normalise the intensity from ^13^C-ethanol. The SEM detector scanned the intensities during two to six independent cycles for each bioreactor and at two different time points.

### Metabolomics

To measure intracellular metabolites, samples were taken at 11 h during the ^13^C-methanol (2%) fermentations, quenched in methanol and frozen at −80 °C. For extraction, the pellet was resuspended in 2 mL of 50% acetonitrile and transferred to 2 mL microcentrifuge tubes with 0.1 mm diameter glass beads, every sample had two technical replicates. Samples were vortexed for 30 s using a Precellys 24 tissue homogeniser (Bertin Instruments) at 30 °C. Three rounds of vortexing were performed allowing the samples to cool completely between rounds. Samples were centrifuged for 3 min at 5000 × *g* and the supernatant was transferred to a clean microcentrifuge tube and stored overnight at −80 °C. In all, 2 mL of samples were freeze-dried overnight, then resuspended in 100 μL of water with 10 µM of AZT as internal standard, then transferred to high-performance liquid chromatography (HPLC) glass inserts for analysis.

Metabolites were analysed using liquid chromatography tandem mass spectrometry as adapted from ref. ^50–52^. In brief, analyses were performed using a Dionex Ultimate 3000 HPLC system coupled to an AB Sciex 4000 QTRAP mass spectrometer. Liquid chromatography was performed using a 50 min gradient, detailed in Supplementary Table 1, with 300 μL/min flowrate, on a Phenomenex Gemini-NX C18 column (150 ×2 mm, 3 μm, 110 A), with a guard column (SecurityGuard Gemini-NX C18, 4 ×2 mm), and column temperature of 55 °C. The mobile phases used were: 7.5 mM aqueous tributylamine (Sigma-Aldrich) with pH adjusted to 4.95 (±0.05) using acetic acid (Labscan) for Solvent A, and acetonitrile (Merck) for Solvent B. Samples were kept at 4 °C in the autosampler and 10 μL were injected for analyses. The HPLC was controlled by Chromeleon 6.80 software (ThermoFisher). Mass spectrometry was achieved using a scheduled multiple reaction monitoring (sMRM) method on the negative ionisation mode. Details of the compound-specific parameters used in the sMRM analysis are listed in Supplementary Table 2. Other hardware parameter values include: ion spray voltage −4500 V, ion source nebuliser (GS1), ion source auxiliary (GS2), curtain (CUR) and collision (CAD) gases were 60, 60, 20 and medium (arbitrary units), respectively, using an in-house ultra-high purity liquid nitrogen tank (BOC). The auxiliary gas temperature was kept at 350 °C. The mass spectrometer was controlled by Analyst 1.6.3 software (AB Sciex). Amounts obtained for each metabolite detected were based on standard curves from serial dilutions of analytical standards, purchased from Sigma, where L1 = 200000, L2 = 100000, L3 = 50000,… L20 = 0.38 nM. This number of standard dilutions guaranteed that the standard curves consisted of at least five data points. Standard mix and pooled samples were regularly injected along the run sequence for quality control. Collected data were processed using MultiQuant 2.1 (AB Sciex).

### Protein extraction and mass spectrometry-based proteomics

Protein samples were taken at 34 h during the ^13^C-methanol (2%) fermentations. Samples were centrifuged at 4 °C 17,000 × *g* for 10 min, the supernatant was discarded and the cell pellet was washed with 1.5 mL of Gibco™ Phosphate-buffered Saline (Life Technologies). Cells were resuspended in lysis buffer (5% SDS, 50 mM triethylammonium bicarbonate, 100 mM DTT, pH 7.55) and transferred to a microcentrifuge tube containing 0.5 mm diameter glass beads. Cells were disrupted by using a Precellys 24 tissue homogeniser (Bertin Instruments) for eight rounds of vortexing (30 s vortex, 45 s rest) without cooling. Microcentrifuge tubes were centrifuged for 10 min at 17,000 × *g* and supernatant was transferred to a clean microcentrifuge tube and stored at −20 °C. Samples were digested with trypsin following the S-trap™ minis (ProtiFi, LCC.) protocol. The digested peptide mixtures were concentrated using Millipore^®^ ZipTip C18 (Merck) eluting with 70% acetonitrile. Residual acetonitrile was removed by vacuum centrifugation and peptides resuspended in 5% acetonitrile, 0.1% formic acid (aqueous) before analysis. Peptides were analysed using a ThermoFisher Scientific UltiMate 3000 RSLCnano UHPLC system. Each sample was initially injected onto a ThermoFisher Acclaim PepMap C_18_ trap reversed-phase column (300 µm × 5 mm nano viper, 5 µm particle size) at a flow rate of 20 µL/min using 2% acetonitrile (aqueous) for 5 min with the solvent going to waste. The trap column was switched in-line with the separation column (ThermoFisher EasySpray Pepmap RSLC C18, 150 µm × 150 mm, 2 µm) and the peptides were eluted using a flowrate of 1.0 µL/min using 0.1% formic acid in water (buffer A) and 80% acetonitrile in buffer A (buffer B) as mobile phases for gradient elution. Peptide elution employed a 4-30% acetonitrile gradient for 40 min followed by 30–50% acetonitrile for 10 min and 50–95% acetonitrile for 1 min at 40 °C. The total elution time was 60 min including a 95% acetonitrile wash followed by re-equilibration. For each sample run, a volume of 2 µL equating to ~1 µg of peptide material from protein digestion was injected.

The eluted peptides from the C_18_ column were introduced to the MS via a nano-ESI and analysed using the Q-Exactive HF-X (ThermoFisher). The electrospray voltage was 1.8 kV in positive ion mode, and the ion transfer tube temperature was 275 °C. Employing a top-40ddMS2 acquisition method, full MS-scans were acquired in the Orbitrap mass analyser over the range m/z 350–1400 with a mass resolution of 120,000 (at m/z 200). The AGC target value was set at 3.00E + 06. The 40 most intense peaks with a charge state between 2 and 5 were fragmented in the high energy collision dissociation cell with a normalised collision energy of 28. MSMS spectra were acquired in the Orbitrap mass analyser with a mass resolution of 15,000 at m/z 200. The AGC target value for MSMS was set to 1.0E + 05 while the ion selection threshold was set to 1E + 03 counts. The maximum accumulation times were 60 min for full MS-scans and MSMS. For all the experiments, the dynamic exclusion time was set to 25 s, and undetermined charge state species were excluded from MSMS.

Raw files were processed using MaxQuant^53^ version 1.6.10.43 with the integrated Andromeda search engine^54^ and the following search parameters: trypsin digestion with a maximum of two missed cleavages, carbamidomethyl (C) as a fixed modification and oxidation (M) and acetyl (protein N-term) as variable modifications. The MS data were searched against the SGD protein sequence database (6630 entries downloaded November 2019). A peptide spectrum match FDR of 0.01 and a protein FDR of 0.01 were used as protein identification level cutoffs. Label-free quantification (LFQ)^55^ was performed using MaxQuant LFQ intensities. Protein quantification analysis of the LFQ results was performed using Perseus^56^ version 1.6.10.0. The data were first filtered to remove identified proteins classified as ‘reverse’, ‘only identified by site’ and ‘potential contaminants’. The two corresponding biological replicates for CEN.PK 113-5D or the reconstructed EC strain were loaded separately and classified as replicates. Proteins that were not present in at least one of the two biological replicates were removed to further trim the data set. All LFQ values were log2 transformed, the median intensity was subtracted to all intensities for all samples to normalise the distribution, and missing values were imputed to 0. Last, a two-sided *t* test was performed between CEN.PK 113-5D and the reconstructed EC strain, and proteins with FDR adjusted *p* values of <0.05 were designated as differentially expressed. The log2FC for the significant proteins was then calculated. A list of all significant proteins that were differentially expressed can be found in Supplementary File 2.

### Statistics and reproducibility

The plotted data are from independent cultures and the spot assays in Fig. 5 were repeated twice with similar results.

### Reporting summary

Further information on research design is available in the Nature Research Reporting Summary linked to this article.

## Supplementary information

Supplementary Information

Supplementary Data 1

Supplementary Data 2

Reporting Summary

Description of Additional Supplementary Files

## Data Availability

All data are included with the published article (and its supplementary information and Source Data files). Raw proteomics data can be downloaded using the following link https://data.mendeley.com/datasets/6n9kdrbcvv/draft?a=6ada27c1-2db7-46e9-83b4-fc34e809cfab. Raw RNA-seq and genome sequencing reads have been deposited at the National Centre for Biotechnology Information under Bioproject number PRJNA612896. Lists of up and downregulated genes were analysed for GO term and pathway enrichment using YeastMine (https://yeastmine.yeastgenome.org/yeastmine/begin.do) at the Saccharomyces Genome Database (https://yeastmine.yeastgenome.org/yeastmine/bag.do). The SGD Protein Sequence database (http://sgd-archive.yeastgenome.org/sequence/S288C_reference/orf_protein/) was used to assign proteomic mass spectra to yeast proteins. Prism 7 software was used to plot data. Any other relevant data are available from the authors upon reasonable request. Source data are provided with this paper.

## Source data

Source Data
