# Supplementary material for: Adaptive laboratory evolution of native methanol assimilation in Saccharomyces cerevisiae
Source: Nat Commun. 2020 Nov 4;11:5564. doi: 10.1038/s41467-020-19390-9 (PMC7643182; doi:10.1038/s41467-020-19390-9)
Supplement: Supplementary file 1 — Supplementary Information [file 41467_2020_19390_MOESM1_ESM.pdf]

Supplementary material for:

Adaptive laboratory evolution of native methanol assimilation in *Saccharomyces cerevisiae*

Monica I. Espinosa [1,2], Ricardo A. Gonzalez-Garcia [3], Kaspar Valgepea [3,4], Manuel Plan [3,5], Colin Scott [2,6], Isak S. Pretorius [1], Esteban Marcellin [3,5], Ian T. Paulsen [1]* and Thomas C. Williams [1,2]*

[1] ARC Centre of Excellence in Synthetic Biology, Department of Molecular Sciences, Macquarie University, NSW, Australia

[2] CSIRO Synthetic Biology Future Science Platform, Canberra, ACT 2601, Australia

[3] Australian Institute for Bioengineering and Nanotechnology, The University of Queensland, St. Lucia, Australia

[4] ERA Chair in Gas Fermentation Technologies, Institute of Technology, University of Tartu, Tartu, Estonia

[5] Metabolomics Australia, AIBN, The University of Queensland, Brisbane, Australia

[6] Biocatalysis and Synthetic Biology Team, CSIRO, Canberra, Australia

* Corresponding authors: tom.williams@mq.edu.au, ian.paulsen@mq.edu.au

Supplementary Table 1. Liquid chromatography gradient profile

Time (min)	% B
0	0
8	0
20	20
30	27
31	100
33	100
34	0
50	0

Supplementary Table 2. Metabolite-specific parameters used in the acquisition of the sMRM data.

Metabolite	Q1 (m/z)	Q3 (m/z)	RT (min)	DP (V)	EP (V)	CE (V)	CXP (V)
PYR	87.02	43.0	12.0	-45	-10	-12	-1
PYR_U13C	90.00	45.0	12.0	-45	-10	-12	-1
LAC	88.95	42.9	8.4	-45	-10	-18	-5
LAC_U13C	92.00	45.0	8.4	-45	-10	-18	-5
FUM	115.01	70.9	20.8	-45	-10	-12	-1
FUM_U13C	119.00	74.0	20.8	-45	-10	-12	-1
SUC	117.01	73.0	18.4	-45	-10	-16	-3
SUC_U13C	121.00	76.0	18.4	-45	-10	-16	-3
OAA	130.93	86.9	20.0	-25	-10	-10	-5
OAA_U13C	135.00	90.0	20.0	-25	-10	-10	-5
MAL	133.00	70.8	19.4	-40	-10	-22	-3
MAL_U13C	137.00	74.0	19.4	-40	-10	-22	-3
KGA	144.95	100.8	20.1	-40	-10	-12	-5
KGA_U13C	150.00	105.0	20.1	-40	-10	-12	-5
PEP	166.83	79.0	21.7	-40	-10	-18	-5
PEP_U13C	170.00	79.0	21.7	-40	-10	-18	-6
GA3P	168.84	97.0	11.8	-40	-10	-10	-5
GA3P_U13C	172.00	97.0	11.8	-40	-10	-10	-5
DHAP	168.84	97.0	11.9	-50	-10	-14	-5
DHAP_U13C	172.00	97.0	11.9	-50	-10	-14	-5
3PG	184.91	97.0	21.1	-50	-10	-20	-5
3PG_U13C	188.00	97.0	21.1	-50	-10	-20	-5
CIT	190.96	110.9	21.8	-50	-10	-18	-7
CIT_U13C	197.00	116.0	21.8	-50	-10	-18	-7
R5P	228.94	96.9	8.7	-20	-10	-30	-15
R5P_U13C	234.00	96.9	8.7	-20	-10	-30	-15
RL5P	228.92	96.9	11.0	-20	-10	-30	-15
RL5P_U13C	234.00	96.9	11.0	-20	-10	-30	-15
G1P	259.02	78.8	9.8	-20	-10	-30	-15
G1P_U13C	265.00	78.8	9.8	-20	-10	-30	-15
G6P	258.89	96.7	8.0	-20	-10	-30	-15
G6P_U13C	265.00	96.7	8.0	-20	-10	-30	-15

F6P	259.02	96.8	9.1	-20	-10	-30	-15
F6P_U13C	265.00	96.8	9.1	-20	-10	-30	-15
F16BP	339.08	96.9	21.4	-20	-10	-30	-15
F16BP_U13C	345.00	96.9	21.4	-20	-10	-30	-15
GLYOX	73.00	45.0	5.7	-45	-10	-12	-1
GLYOX_U13C	75.00	46.0	5.7	-45	-10	-12	-1
GLYCO	75.00	47.0	5.8	-35	-10	-14	-3
GLYCO_U13C	77.00	48.0	5.8	-35	-10	-14	-3
UDPG	565.18	323.0	20.8	-90	-10	-34	-7
UDPG_U13C	571.00	323.0	20.8	-90	-10	-34	-7
UDPGA	579.14	79.1	28.6	-90	-10	-108	-1
UDPGA_U13C	585.00	79.1	28.6	-90	-10	-108	-1
ACOA	808.17	79.1	32.2	-125	-10	-54	-5
ACOA_U13C	810.00	79.1	32.2	-125	-10	-54	-5
UDPNAc	605.86	78.7	20.9	-95	-10	-106	-1
UDPNAc_U13C	612.00	78.7	20.9	-95	-10	-106	-1
ACO	172.94	84.9	22.0	-30	-10	-18	-5
ACO_U13C	177.00	87.0	22.0	-125	-10	-54	-5
CMP	322.07	78.8	11.9	-65	-10	-66	-3
UMP	323.01	78.8	13.4	-60	-10	-64	-3
AMP	346.02	78.6	15.9	-70	-10	-62	-3
GMP	362.05	78.9	14.1	-60	-10	-62	-3
UDP	403.03	78.8	21.5	-60	-10	-74	-3
ADP	426.07	78.8	22.0	-85	-10	-74	-3
GDP	442.06	78.9	21.3	-70	-10	-76	-3
CTP	481.95	158.6	27.8	-75	-10	-36	-11
UTP	483.06	158.8	29.1	-65	-10	-42	-7
ATP	506.10	158.7	29.4	-85	-10	-40	-11
GTP	522.00	158.7	28.8	-80	-10	-42	-11
NAD	662.25	540.0	13.3	-50	-10	-20	-9
NADH	664.20	78.8	22.3	-110	-10	-98	-1
NADP	742.20	620.0	21.2	-45	-10	-24	-11
NADPH	744.10	79.1	29.2	-120	-10	-116	-1
Cre-P	209.74	78.8	19.0	-35	-10	-16	-3
AZT	265.80	223.0	14.0	-70	-10	-16	-1

Note: U13C: denotes ^{13}C universally labelled metabolite, Q, quadrupole; RT, retention time; DP, declustering potential; EP, entrance potential; CE, collision energy; CXP, collision cell exit potential; V, volts.

Supplementary Table 3. Primers used in this study.

Primer	Name	5' to 3' sequence (annealing regions in upper case)
1	acs1Δ F	ATCACATGTGCACATACGTCC
2	acs1Δ R	AATGAGCAAGTCGATACAAGG
3	adh2Δ F	GGCAAACAAACGGAAAAATCG
4	adh2Δ R	TCTACGGAACCCTGATCAAGC
5	shm1Δ F	GAAAGCTCGCTAATTCTTACC
6	shm1Δ R	CGTTCATCACCAAGACAATCG
7	cat8Δ F	TTTCTATCCGGTGTTTATTCG
8	cat8Δ R	TAACCATGGCAATACTATAGC
9	acs1Δ check F	GGGTATAAATGTTTTCCAAGG
10	acs1Δ check R	CAAGAACATTAGGAATAGTGG
11	adh2Δ check F	CGAAATCACCAATTCTAAACC
12	adh2Δ check R	TGTACCACAGTGTTGGATTCC
13	shm1Δ check F	GTATTTGATATGGGAATCTGC
14	shm1Δ check R	GAATACGATATGGAAGCAACC
15	cat8Δ check F	GATAAATAAGGAGGGCAATGC
16	cat8Δ check R	TCACCTCTTCATCTTCTGTGG
17	yMCc-crRNA F	attataaaatacacgtacgGTTTTAGAGCTAGAAATAGCAAGTTA
18	yMCc-crRNA R	cgtacgtgtattttataataGATCATTTATCTTTCACTGCG
19	YGR067C F	GCAGGTTAAAAGCCGGTTGG
20	YGR067C R	CTGGGGTGGGACCACTATTG
21	YGR067C check F	GTCTTACGTGACAGGGTCCG
22	YGR067C check R	TTCTTCCCATGATATTCAGTGATGT
