# Supplementary material for: Adaptive laboratory evolution of native methanol assimilation in Saccharomyces cerevisiae
Source: Nat Commun. 2020 Nov 4;11:5564. doi: 10.1038/s41467-020-19390-9 (PMC7643182; doi:10.1038/s41467-020-19390-9)
Supplement: Supplementary file 4 — Reporting Summary [file 41467_2020_19390_MOESM4_ESM.pdf]

nature research

Corresponding author(s): Tom Williams and Ian Paulsen

Last updated by author(s): Oct 1, 2020

Reporting Summary

Nature Research wishes to improve the reproducibility of the work that we publish. This form provides structure for consistency and transparency in reporting. For further information on Nature Research policies, see our Editorial Policies and the Editorial Policy Checklist.

Statistics

For all statistical analyses, confirm that the following items are present in the figure legend, table legend, main text, or Methods section.

n/a	Confirmed	
☐	☒	The exact sample size (*n*) for each experimental group/condition, given as a discrete number and unit of measurement
☐	☒	A statement on whether measurements were taken from distinct samples or whether the same sample was measured repeatedly
☐	☒	The statistical test(s) used AND whether they are one- or two-sided<br>*Only common tests should be described solely by name; describe more complex techniques in the Methods section.*
☒	☐	A description of all covariates tested
☐	☒	A description of any assumptions or corrections, such as tests of normality and adjustment for multiple comparisons
☐	☒	A full description of the statistical parameters including central tendency (e.g. means) or other basic estimates (e.g. regression coefficient) AND variation (e.g. standard deviation) or associated estimates of uncertainty (e.g. confidence intervals)
☐	☒	For null hypothesis testing, the test statistic (e.g. *F*, *t*, *r*) with confidence intervals, effect sizes, degrees of freedom and *P* value noted<br>*Give P values as exact values whenever suitable.*
☒	☐	For Bayesian analysis, information on the choice of priors and Markov chain Monte Carlo settings
☒	☐	For hierarchical and complex designs, identification of the appropriate level for tests and full reporting of outcomes
☒	☐	Estimates of effect sizes (e.g. Cohen's *d*, Pearson's *r*), indicating how they were calculated

Our web collection on statistics for biologists contains articles on many of the points above.

Software and code

Policy information about availability of computer code

Data collection	HPLC was controlled by Chromeleon 6.80 software (ThermoFisher). For metabolomics, the mass spectrometer was controlled by Analyst 1.6.3 software (AB Sciex).
Data analysis	Geneious Pro version 11 was used to analyse genome and transcriptome sequencing data. Graphpad Prism version 7 was used to plot and statistically test growth and metabolite concentration data. For proteomics, raw files were processed using MaxQuant version 1.6.10.43 with the integrated Andromeda search engine. Protein quantification analysis of label free quantification results was performed using Perseus version 1.6.10.0. Metabolomics data were processed using MultiQuant 2.1 (AB Sciex).

For manuscripts utilizing custom algorithms or software that are central to the research but not yet described in published literature, software must be made available to editors and reviewers. We strongly encourage code deposition in a community repository (e.g. GitHub). See the Nature Research guidelines for submitting code & software for further information.

Data

Policy information about availability of data

All manuscripts must include a data availability statement. This statement should provide the following information, where applicable:

- Accession codes, unique identifiers, or web links for publicly available datasets
- A list of figures that have associated raw data
- A description of any restrictions on data availability

All data are included in the published article (and its supplementary information and Source Data files). Raw proteomics data can be downloaded using the following link https://data.mendeley.com/datasets/6n9kdrbcvv/draft?a=6ada27c1-2db7-46e9-83b4-fc34e809cfab. Raw RNA-seq and genome sequencing reads have been deposited at the National Centre for Biotechnology Information under Bioproject number PRJNA612896. Lists of up and down-regulated genes were analysed for GO term and pathway enrichment using YeastMine (https://yeastmine.yeastgenome.org/yeastmine/begin.do) at the Saccharomyces Genome Database (https://

yeastmine.yeastgenome.org/yeastmine/bag.do). The SGD Protein Sequence database (http://sgd-archive.yeastgenome.org/sequence/S288C_reference/orf_protein/) was used to assign proteomic mass spectra to yeast proteins. Prism 7 software was used to plot data. Source data are provided with this paper.

Field-specific reporting

Please select the one below that is the best fit for your research. If you are not sure, read the appropriate sections before making your selection.

- [x] Life sciences
- [ ] Behavioural & social sciences
- [ ] Ecological, evolutionary & environmental sciences

For a reference copy of the document with all sections, see nature.com/documents/nr-reporting-summary-flat.pdf

Life sciences study design

All studies must disclose on these points even when the disclosure is negative.

Sample size	No sample size calculation was performed. Sample size was chosen based on experimental feasibility with bioreactor set-ups and 13C-methanol cost. Biological duplicates are appropriate when conditions are well-controlled in bioreactors and strain replicates are genetically identical, and are commonly used in the field of metabolic engineering.
Data exclusions	No data were excluded
Replication	A minimum of biological duplicates were used for all experiments. 13C metabolomics, transcriptomics, proteomics, and OD600 growth profiling were conducted using two independent biological replicates in bioreactors. Experiments on methanol dependent growth of wild-type and evolved-reconstructed yeast were conducted in two different labs, at Macquarie University in shake-flasks (Figure 2 c), and at The University of Queensland in bioreactors (Figure 3 a). Adaptive Laboratory Evolution was carried out using three independent lineages. All attempts at replication were successful.
Randomization	Yeast strains were allocated into experimental groups based on their genotypes and/or growth medium. Sample order was randomised for metabolomics data collection. Control of co-variates is not relevant as all other conditions/variables were experimentally controlled.
Blinding	Investigators were blind to sample identity during metabolomics data collection and analysis. Blinding was otherwise not possible or relevant, as group allocation information was necessary to conduct the experiments.

Reporting for specific materials, systems and methods

We require information from authors about some types of materials, experimental systems and methods used in many studies. Here, indicate whether each material, system or method listed is relevant to your study. If you are not sure if a list item applies to your research, read the appropriate section before selecting a response.

Materials & experimental systems

n/a	Involved in the study	
x		Antibodies
	x	Eukaryotic cell lines
x		Palaeontology and archaeology
x		Animals and other organisms
x		Human research participants
x		Clinical data
x		Dual use research of concern

Methods

n/a	Involved in the study	
x		ChIP-seq
x		Flow cytometry
x		MRI-based neuroimaging

Eukaryotic cell lines

Policy information about cell lines

Cell line source(s)	CEN.PK113-5D was sourced from the EUROSCARF collection.
Authentication	Our strain of CEN.PK113-5D was verified using whole-genome re-sequenced as part of another study in our lab.
Mycoplasma contamination	Cell lines were not tested for mycoplasma contamination.
Commonly misidentified lines (See ICLAC register)	No commonly misidentified cell lines were used in this study.
