# Supplementary material for: Adaptive laboratory evolution of native methanol assimilation in Saccharomyces cerevisiae
Source: Nat Commun. 2020 Nov 4;11:5564. doi: 10.1038/s41467-020-19390-9 (PMC7643182; doi:10.1038/s41467-020-19390-9)
Supplement: Supplementary file 5 — Description of Additional Supplementary Files [file 41467_2020_19390_MOESM5_ESM.pdf]

Title: Supplementary Data 1:
Description: Processed transcriptome data Gene names, fold changes, and significance levels are listed for the Reconstructed Evolved C strain relative to the control CEN.PK113-5D strain when grown in yeast extract methanol medium.

Title: Supplementary Data 2:
Description: Processed proteome data All proteins that were significantly differentially expressed are listed. Label free quantitation (LFQ) values, significance levels, the number of peptides identified, and protein IDs are listed for the two biological replicates of the control CEN.PK113-5D and the Reconstructed Evolved C strain.
